# Cross-cultural adaptation and validation of the Italian version of the Western Ontario Rotator Cuff (WORC) index

**DOI:** 10.1007/s12306-023-00812-y

**Published:** 2024-01-29

**Authors:** G. Milano, L. Fresta, S. Cerciello, S. Cattaneo, M. Paderno, C. Galante, M. Passiatore, M. F. Saccomanno

**Affiliations:** 1 Department of Medical and Surgical Specialties, Radiological Sciences, and Public Health, University of Brescia, Brescia, Italy; 2 Unit of Orthopaedics, Department of Bone and Joint Surgery, ASST Spedali Civili, Piazzale Spedali Civili, 1, 25123 Brescia, BS Italy; 3 Department of Orthopaedics and Traumatology, San Carlo di Nancy Hospital, Rome, Italy; 4 Department of Orthopaedics and Traumatology, Fondazione Policlinico Universitario Agostino Gemelli IRCCS, Rome, Italy

**Keywords:** Rotator cuff, Patient-reported outcome measures, Validity, Cross-cultural adaptation, Quality of life

## Abstract

The aim of the study was to accomplish translation, cross-cultural adaptation and validation of the Western Ontario Rotator Cuff (WORC) Index questionnaire for its use in Italy. The WORC original version was translated and cross-culturally adapted into Italian. Subsequently, it was administered to a population of 60 patients suffering from rotator cuff disease to evaluate the validity and reliability of the Italian version. The content validity evaluated the correlation between questions and total score of each domain through Pearson’s correlation coefficient. The construct validity was similarly assessed through Pearson’s correlation coefficient by testing the correlation between the Italian WORC and the Italian version of the Disability of the Arm, Shoulder and Hand (DASH) questionnaire. Reliability was assessed using two methods: internal consistency by calculating the Cronbach’s alpha coefficient for each domain; and test–retest by means of the intraclass correlation coefficient (ICC). The translation and cross-cultural adaptation of the Italian version did not reveal any major problems. No significant floor or ceiling effects were found. All the questions were linearly related to the concept expressed by the domain of belonging. Overall correlation with the DASH score was 0.75. Internal consistency was very high overall (*α* = 0.93) as well as reliability (overall ICC = 0.87). The Italian version of the WORC questionnaire is a valid and reproducible measuring instrument and can be considered a valid tool for the evaluation of the effectiveness of a treatment in terms of quality of life, in Italian patients affected by rotator cuff diseases.

*Level of evidence* Diagnostic study, level II.

**Supplementary Information:**

The online version contains supplementary material available at 10.1007/s12306-023-00812-y.

## Introduction

Rotator cuff (RC) disorders contain a wide spectrum of pathologic conditions, ranging from subacromial impingement to full-thickness tears, which results in pain, functional disability, reduction in work and sports activities, thus affecting the quality of life (QOL) [1].

Disease-specific QOL questionnaires, as the name suggests, tend to measure more specific elements of the respective disease and are theoretically more sensitive to individual’s perception of treatment-related changes than generic QOL measures [2].

Patient-reported outcome measures (PROMs) seek to quantify patients’ outcomes and are nowadays integral part of the orthopedic practice. It must be noticed that over the last decade, self-administered PROMs grew at higher rates than their clinician-dependent counterparts, highlighting an emphasis on patients’ perspective [3].

Several shoulder functional and subjective outcome assessment tools are available [4, 5]. In 2003, Kirkley et al. [6] introduced the Western Ontario Rotator Cuff (WORC) Index, a self-administered disease-specific questionnaire that can assess QOL in patients with RC disease.

Although there is no questionnaire intended as a gold standard for the study of RC disorders, WORC has been applicable in most cases. Moreover, it allows stratifying the results in subdomains, thus providing a more detailed characterization of the state of illness. The monitoring of the results obtained in this way provided a complete evaluation of the effects of the treatment, adapting this tool to the modern concept of health and QOL [7].

The validity, reliability and responsiveness of the WORC Index questionnaire have been widely demonstrated in the literature [6, 8–15]. Moreover, it has been translated and validated in several languages [16–26]. However, there is no version translated and validated in Italian so far.

The purpose of the present study was to accomplish translation, cross-cultural adaptation and validation of the WORC Index questionnaire for its use in Italy. The hypothesis of the study is that the Italian national version of the WORC questionnaire is as valid as the original version.

## Methods

The study was divided into two phases. In the first phase, the process of translation and cross-cultural adaptation of the questionnaire from the original American English to Italian version was carried out [27, 28]. Subsequently, the WORC was administered to a population of patients suffering from rotator cuff disease to evaluate the validity of the translated final version [7, 29–31].

The study was approved by the local Ethic Committee and all patients involved signed an informed consent to enter the study.

### Translation and cross-cultural adaptation

The WORC questionnaire was translated according to the guidelines for the cross-cultural adaptation process of the America Association of Orthopedic Surgeons (AAOS) Outcomes Committee [27]. The translation process has been divided into different phases:

#### Initial translation

Two bilingual translators (Italian and American English) independently translated the questionnaire from American English to Italian language. One of the two translators was a fellowship-trained shoulder surgeon, the other a professional translator. Both proposed a more than literal conceptual translation of the text of the questionnaire and in a colloquial language suitable for readers of 14 years of age.

#### Summary of the translation

The two translators, assisted by the project’s scientific coordinator (MS), compared the two translations to arrive at a common version (version 1). The points of disagreement were resolved by discussion.

#### Back-translation into the original language

Two native American English language professional translators with no medical skills, nor aware of the original version of the questionnaire, independently translated version 1 into American English. The aim of this back-translation was to make sure that the contents of the questionnaire were not altered by the translation in a language different from the original one.

#### Review by an ad hoc commission

A committee of experts then compared the two translations of version 1 into American English with the original questionnaire to obtain the trans-cultural adaptation and the pre-final version of the translated questionnaire. This commission was composed of four translators, the senior author, two epidemiologists, a statistician and an Italian linguist. Every disagreement has been resolved through discussion and consensus.

#### Test of the pre-final version

The Italian pre-final version was tested on 30 patients suffering from RC diseases. After completing the questionnaire, each patient was questioned by a physician or a therapist about any difficulties in compiling the same or understanding the meaning of each question and each answer. After the interviews, the committee of experts discussed what emerged in order to obtain a final version. At the end of each phase, a detailed report was produced that highlighted the difficulties of the translation process and motivated the rationale of all the choices.

### Study population

The second part of the study was conducted on a sample population suffering from RC disease, including RC tendinopathy, partial or full-thickness tears, to evaluate the validity of the translated final version.

All patients with shoulder pain were considered eligible for the study. Diagnosis of rotator cuff disease was confirmed at clinical and instrumental evaluation in order to assess their eligibility for the study. The diagnostic imaging consisted of impingement x-ray series (true anteroposterior, axillary and outlet views) and magnetic resonance imaging (MRI) of the affected shoulder.

Patients who satisfied the following criteria were included in the study: diagnosis of tendonitis, partial-thickness tears, full-thickness tears, or calcific tendonitis of the rotator cuff, as assessed at radiologic exams, pain in at least 2 (out of 3) of abduction, external rotation and internal rotation isometric tests, positive impingement tests and acceptance to enter the study.

Patients with the following characteristics were excluded from the study: diagnosis of capsulolabral pathology (glenohumeral instability, SLAP lesions), adhesive capsulitis, glenohumeral osteoarthritis (OA), previous fractures or surgical procedures to the same shoulder, clinical signs and symptoms of cervical radiculopathy, neurological disease involving upper limbs (e.g., axillary nerve injury, syringomyelia), infectious disease, rheumatic disease, potential worker’s compensation syndrome, or inability to complete the questionnaires for linguistic or cognitive problems.

### Intervention

Patients enrolled for the study were given simultaneously the WORC questionnaire and the Italian version of the questionnaire Disability of the Arm, Shoulder and Hand (DASH) [32].

The WORC questionnaire is a self-administered pathology-specific tool for assessing quality of life in patients with rotator cuff disease [6]. It consists of 21 questions, divided into 5 domains: physical symptom (6 questions), sports/recreation (4 questions), work (4 questions), lifestyle (4 questions), and emotions (3 questions). The score of each question is evaluated on the VAS scale from 0 (minimum disability, best result) to 100 points (maximum disability, worst result). The overall score therefore varies from 0 to 2100, where a higher score is indicative of a worse quality of life. By means of arithmetic conversion, the total score is expressed as a percentage.

The DASH score is a 30-item self-administered questionnaire concerning the patient’s health status. The items explore the difficulties in performing different physical tasks (21 items), the severity of symptoms (5 items), as well as the functional impact on social activities, work and sleep (4 items). The scoring system of the questionnaire is based on a metric scale, ranging from 0 (minimum disability, best result) to 100 points (maximum disability, poorest result).

The questionnaires WORC and DASH were completed by each patient at the time of enrollment. The final translated version of the WORC questionnaire was again submitted to each patient after 4 weeks from the previous (retest). To minimize the risk of any change in the clinical condition, no patients underwent any treatment before retest.

### Outcome measures

The validation of the translated final version was carried out in accordance with the analysis plan suggested by the IQOLA project [29, 31]. Validation consisted in verifying the following properties of the final version:

#### Content validity

Testing of hypotheses that are the basis of the construction of the tool’s questions through a series of psychometric tests that assess the adequacy of the questions and the correlation between the questions and the domains within which they are included.

#### Construct validity

Testing of the correlation between the translated final version and other questionnaires that evaluate the same hypotheses.

#### Reliability

Testing of the reproducibility of the questionnaire.

### Data analysis

All data were analyzed using IBM SPSS Statistics 25 software (IBM, Armonk, NY, USA). The descriptive statistics included two analysis levels:

#### Item level (questions)

Percentage and distribution of missing data; mean and standard deviation of each question have been reported. Distribution of responses for each question in each domain has been explored and the Shapiro–Wilk test was used to verify data normality.

#### Scale level (domains)

Evaluation of mean and standard deviation of each domain have been reported. Ratio between observed minimum values and minimum possible values, and ratio between observed maximum values and maximum possible values were explored to assess ceiling and floor effect. These effects were confirmed if more than 15% of the patients achieved the highest or the lowest possible score [30, 33].

The content validity was evaluated through multi-trait analysis, which is a psychometric technique used to examine the correlation between each question and its hypothetical domain, as well as the correlation between each question and the other domains. It allows to test the following hypotheses at the same time:

#### Item internal consistency

Linear correlation between questions and total score of each domain. The correlation was evaluated by Pearson’s correlation coefficient. Correlation coefficient (corrected for overlap) was scored as follows: very weak between 0 and 0.19; weak between 0.20 and 0.39, moderate between 0.40 and 0.69; strong between 0.70 and 0.89; very strong between 0.90 and 1 [34].

#### Equality of item-scale correlation

All questions within the same domain should contribute proportionally in the same way to the total score of the domain. Therefore, for questions in the same domain, the correlation between each question and the sum of the score of the others should be similar for all the questions.

#### Item discriminant validity

Each question should have a significantly higher correlation with its own domain than with other domains that evaluate different concepts. Correlation was considered significant if greater than two standard errors.

The construct validity was assessed by testing the correlation between the translated final version of the WORC questionnaire and the translated Italian version of the DASH questionnaire. The Pearson’s coefficient was used to test this correlation and scored as follows: very weak between 0 and 0.19; weak between 0.20 and 0.39, moderate between 0.40 and 0.69; strong between 0.70 and 0.89; very strong between 0.90 and 1.00, showing a strong range in all domains [34].

Reliability was assessed using two methods: internal consistency and test–retest. Internal consistency was evaluated by calculating the Cronbach’s alpha coefficient for each domain. The minimum acceptable reliability coefficient for patient’s individual score is 0.70, which indicates a good reliability [35]. Test–retest reliability was evaluated by means of the intraclass correlation coefficient (ICC 1, 2 model). ICC values ranged from 0 to 1, with 1 indicating perfect reliability, and they were interpreted as follows: values less than 0.50 are indicative of poor reliability; values between 0.50 and 0.75 indicate moderate reliability; values between 0.75 and 0.90 indicate good reliability, and values greater than 0.90 indicate excellent reliability [36].

### Sample size calculation

As long as there are no absolute rules for the sample size needed to validate a questionnaire, in accordance with Altman [37] and Terwee et al. [30], the study should include a minimum sample of 50 patients. We increased this number to 60 to compensate a potential 20% drop-out at recall for assessment of test–retest reliability.

## Results

### Translation and trans-cultural adaptation

The translations of the questionnaire from the original language to Italian and vice versa from version 1 to the English American did not reveal any major problems or difficulties with language. All parts of the questionnaire were translated without difficulty. All the interviewed patients understood the translated questions well and answered without difficulty. The completion of the questionnaire took approximately 10 min for each patient (Online appendix 1).

### Study population

The study population consisted of 60 patients, including 23 males (38.3%) and 37 females (61.7%). The age was between 26 and 88 years (mean age: 61 ± 11.5 years), the dominance of the affected side was observed in 42 cases (70%).

### Descriptive statistics (item level)

The descriptive statistics data are shown in Table 1. To confirm the understanding of the translated questionnaire, there are no missing data. Wide response ranges were observed. Being a population of patients suffering from shoulder disorders, the distribution of responses in most domains was always directed toward higher (worse) scores. Only the questions A-5 (about “the presence of clicks or crackles in the shoulder”) and A-6 (about “the pain felt by the neck muscles due to the shoulder”) showed a distribution of responses to lower scores. The Shapiro–Wilk test confirmed normal distribution of data for each domain.

**Table 1:** Descriptive statistics for items

Items	Missing (%)	Mean	SD	Normality test (*p*-value)
*Scale A (Physical symptoms)*				
A-1	0.0	60.35	25.12	0.002
A-2	0.0	51.02	28.34	0.005
A-3	0.0	53.05	26.99	0.028
A-4	0.0	55.74	27.52	0.007
A-5	0.0	47.40	33.83	< 0.0001
A-6	0.0	48.06	33.47	< 0.0001
*Scale B (Sports/recreation)*				
B-1	0.0	60.44	29.07	< 0.0001
B-2	0.0	70.82	28.54	< 0.0001
B-3	0.0	64.81	31.48	< 0.0001
B-4	0.0	51.32	37.12	< 0.0001
*Scale C (Work)*				
C-1	0.0	61.29	28.65	< 0.0001
C-2	0.0	67.29	27.38	< 0.0001
C-3	0.0	68.72	30.74	< 0.0001
C-4	0.0	68.31	30.41	< 0.0001
*Scale D (Lifestyle)*				
D-1	0.0	61.23	29.20	0.003
D-2	0.0	52.02	33.48	0.001
D-3	0.0	63.74	34.96	< 0.0001
D-4	0.0	59.74	31.71	< 0.0001
*Scale E (Emotions)*				
E-1	0.0	54.19	29.85	0.002
E-2	0.0	49.97	32.94	< 0.0001
E-3	0.0	56.87	34.47	< 0.0001

### Descriptive statistics (scale level)

Tables 2 and 3 show, for raw and normalized data, mean and standard deviation of each domain, minimum and maximum values and range, and proportion of lowest (floor) and highest (ceiling) responses. The answers used the visual-analogue scale extensively; the floor effects were not found except for 4.8% in section E; the ceiling effects was observed for 3.2% in sections B and C only; for the remaining sections, the floor and ceiling effects were 0.

**Table 2:** Descriptive statistics for scales (raw scores)

Scale	Observed/possible values						
	Mean	SD	Lowest	Highest	Range	% at floor	% at ceiling
A	315.6	137.5	30/0	560/600	530/600	0	0
B	247.4	109.4	20/0	400/400	380/400	0	3.2
C	265.6	101.8	15/0	400/400	385/400	0	3.2
D	236.7	111.1	10/0	395/400	385/400	0	0
E	161	88.8	0/0	290/300	290/300	4.8	0

**Table 3:** Descriptive statistics for scales (normalized scores)

Scale	Transformed scores: 0–100						
	Mean	SD	Lowest	Highest	Range	% at floor	% at ceiling
A	52.6	22.9	5/0	93/100	88/100	0	0
B	61.9	27.4	5/0	100/100	95/100	0	3.2
C	66.4	25.4	4/0	100/100	96/100	0	3.2
D	59.2	27.8	3/0	99/100	96/100	0	0
E	53.7	29.6	0/0	97/100	97/100	4.8	0

### Item internal consistency

All the correlations between each question in a given domain and the scores of the other questions in the same domain (corrected for overlap) showed Pearson’s coefficient greater than 0.40 showing a more than acceptable correlation (Table 4). In section A, values from 0.664 and 0.858 were observed while in section B values between 0.824 and 0.907; in section C values between 0.803 and 0.901; in section D values between 0.773 and 0.919; in section E, there were the highest scores between 0.898 and 0.936.

**Table 4:** Item internal consistency

Items	Scales				
	A	B	C	D	E
*Scale A (Physical symptoms)*					
A-1	0.759*	0.589	0.604	0.591	0.584
A-2	0.841*	0.683	0.574	0.680	0.698
A-3	0.793*	0.731	0.616	0.722	0.713
A-4	0.799*	0.722	0.695	0.775	0.553
A-5	0.664*	0.437	0.361	0.471	0.442
A-6	0.858*	0.657	0.721	0.825	0.762
*Scale B (Sports/recreation)*					
B-1	0.745	0.889*	0.659	0.782	0.622
B-2	0.776	0.836*	0.587	0.615	0.481
B-3	0.726	0.907*	0.703	0.780	0.626
B-4	0.691	0.824*	0.766	0.799	0.766
*Scale C (Work)*					
C-1	0.785	0.799	0.901*	0.833	0.672
C-2	0.636	0.771	0.901*	0.750	0.508
C-3	0.381	0.488	0.803*	0.612	0.374
C-4	0.623	0.717	0.874*	0.779	0.565
*Scale D (Lifestyle)*					
D-1	0.699	0.699	0.634	0.773*	0.521
D-2	0.725	0.715	0.781	0.9*	0.618
D-3	0.637	0.763	0.698	0.838*	0.626
D-4	0.820	0.804	0.816	0.919*	0.717
*Scale E (Emotions)*					
E-1	0.766	0.724	0.553	0.673	0.906*
E-2	0.709	0.615	0.490	0.629	0.936*
E-3	0.657	0.677	0.618	0.682	0.898*

*Values corrected for overlap

### Equality of item-scale correlation

A higher range of question-domain correlation was observed for section E “Emotions”. However, the questions in each domain contribute equivalently to the total score of the domain.

### Item discriminant validity

The correlation between the question and the domain to which it belonged was significantly higher than the correlation between the same question and the other domains in all cases (Table 5). Percentages of questions that showed a higher correlation with the domain of belonging than with the other domains ranged between 91.7% and 100% for section A, between 93.8% and 100% for section B and equal to 100% for the remaining three sections (Table 6).

**Table 5:** Item-scale discriminant validity tests

Items	Scales				
	A	B	C	D	E
*Scale A (Physical symptoms)*					
A-1	–	2	2	2	2
A-2	–	2	2	2	2
A-3	–	2	2	2	2
A-4	–	2	2	1	2
A-5	–	2	2	2	2
A-6	–	2	2	1	2
*Scale B (Sports/recreation)*					
B-1	2	–	2	2	2
B-2	2	–	2	2	2
B-3	2	–	2	2	2
B-4	2	–	2	1	2
*Scale C (Work)*					
C-1	2	2	–	2	2
C-2	2	2	–	2	2
C-3	2	2	–	2	2
C-4	2	2	–	2	2
*Scale D (Lifestyle)*					
D-1	2	2	2	–	2
D-2	2	2	2	–	2
D-3	2	2	2	–	2
D-4	2	2	2	–	2
*Scale E (Emotions)*					
E-1	2	2	2	2	–
E-2	2	2	2	2	–
E-3	2	2	2	2	–

**Table 6:** Frequency and percentage of Item-scale correlations at each level of scaling process

Scale	− 2		− 1		1		2		1 + 2	
	*n*	*%*	*n*	*%*	*n*	*%*	*N*	*%*	*n*	*%*
A	0	0	0	0	2	8.3	22	91.7	24	100
B	0	0	0	0	1	6.2	15	93.8	16	100
C	0	0	0	0	0	0	16	100	16	100
D	0	0	0	0	0	0	16	100	16	100
E	0	0	0	0	0	0	12	100	12	100

### Construct validity

Correlation analysis between the Italian WORC and the DASH questionnaire showed an overall correlation coefficient of 0.749, with a significant correlation between each domain of the WORC and the DASH questionnaire (Table 7).

**Table 7:** Correlation between WORC and another measurement tool (DASH evaluation form)

Scales	DASH	
	Perason’s coefficient	*p*-value
A	0.659	0.000
B	0.676	0.000
C	0.626	0.000
D	0.770	0.000
E	0.658	0.000
Overall	0.749	0.000

### Reliability

Internal consistency was calculated using Cronbach’s alpha, with data obtained from the administration of the questionnaire to all patients (Table 8). The internal consistency of the translated final version of the WORC questionnaire was > 0.90 in all domains except for section E, where coefficient was equal to 0.831.

**Table 8:** Reliability coefficients and inter-scale correlations

Scale	A	B	C	D	E	Overall
A	0.919					
B	0.804	0.954				
C	0.693	0.794	0.910			
D	0.836	0.868	0.854	0.921		
E	0.776	0.734	0.608	0.725	0.831	
Overall	0.921	0.930	0.871	0.950	0.841	0.929

Scale internal consistency reliability (Cronbach’s alpha) is presented in the diagonal

The test–retest was conducted on a sample of 40 patients who completed the questionnaire a second time, after a period of two weeks after the enrollment. The ICCs are shown in Table 9. The ICC values were between 0.72 and 0.92 for section A (physical symptoms), between 0.83 and 0.96 for section B (sport), between 0.67 and 0.91 for section C (work), between 0.7 and 0.92 (lifestyle), and between 0.49 and 0.85 for section E (emotions). The overall reliability of the WORC was equal to 0.87, which is almost excellent.

**Table 9:** Intraclass correlation coefficients

Scales	ICC	95%CI	
		Lower limit	Upper limit
A	0.85	0.72	0.92
B	0.91	0.83	0.96
C	0.83	0.67	0.91
D	0.85	0.7	0.92
E	0.72	0.49	0.85
Overall	0.87	0.75	0.93

## Discussion

The main finding of the present paper is that the Italian translation of the WORC questionnaire respected the format of the original version while using a simple and immediate vocabulary, so it was immediately accessible and could be easily understood by each patient. Moreover, the Italian version showed very good psychometric properties. The multi-trait analysis showed that all the questions are linearly related to the concept expressed by the domain of belonging, each question within each domain contributes proportionally to the score of the domain, and each question correlates better with its own domain compared to the other domains. Even if small floor and ceiling effects were found in some sections, they are considered to be significant only if more than 15% of respondents achieved the lowest or highest possible score, respectively [30]. Since it was not the case, it can be said that content validity was appropriate, which means that patients with the lowest or highest possible score could be distinguished from each other.

The internal consistency, assessed by using Cronbach’s alpha, was found to be 0.93, with the range for individual domains from 0.83 to 0.95. Cronbach’s alpha of at least 0.70 has been suggested to indicate adequate internal consistency [38]; therefore, the present results confirm excellent internal consistency for the Italian version. On the other hand, it must be also taken into account that it is not always the more the better, since values higher than 0.90 could suggest that some items may be redundant [38]. Unfortunately, this could be a bias already present in the original version, since results of the present study are consistent with results of the original (*α* = 0.93) [6] and other translated versions (range *α* = 0.68–0.97) [16–26]. In this perspective, a shorten version of the original WORC has been recently proposed [11]. It contains only seven of the original 21 questions, including all items from the WORC work and lifestyle domains except the one relating to roughhousing. The internal consistency of short-version WORC, assessed by using Cronbach’s alpha, was found to be 0.89, therefore lower and more acceptable compared to the original version [6]. However, analysis of a “short” Italian version was beyond the aim of the study and therefore, was not conducted.

Test–retest reliability of the Italian version also showed excellent results with an overall score of 0.87 and range for individual domains between 0.72 and 0.91. Since a heterogeneous group of patients was included, it is important to remember that the ICC is highly dependent on the variation of the study sample. The recommended ICC for an assessment tool is > 0.7 for a large group (as in research) or > 0.9 for individuals [39]. Therefore, the results of this study indicate that the Italian version has adequate reliability for use in a large group. The present results are comparable to the original questionnaire (ICC = 0.96) [6] and other translations (range ICC = 0.74–0.99) [16–26].

Finally, correlations between the WORC domains and the Italian version of the DASH also proved to be strong as expected, accordingly with the original [6] and other translated versions [16, 17, 19–23, 26].

In the last decade, the way to perceive the concept of health and to evaluate the results of a treatment has somehow changed. Collection of patient-centric quality metrics continues to grow in importance as health care moves in the direction of a value-based system. Therefore, subjective assessment on the results of a treatment or on the influence of clinical results has assumed a fundamental importance, equal or superior to the objective clinical evaluation. A recent systematic review [40] which evaluated the cross-cultural adaption procedures and measurement properties reported in 14 published translated versions of the WORC questionnaire confirmed that it is the superior choice of PROMs for evaluating RC pathology, regardless of culture. This assumption surely adds importance and value to the present study. The increasing necessity to conduct international research projects and to compare results of the same treatments in various parts of the world makes indispensable to use the same assessment systems worldwide, and therefore a correct translation and validation of the measurement systems become of utmost importance rather than the hypothesis to formulate new questionnaires. As correctly highlighted by guidelines and recent studies [27, 40], correct translation does not provide for a literal translation of each word, but a cultural adaptation of the proposed items in order to maintain the psychometric properties of the evaluation system in question unaltered. Şahinoğlu et al. [12] recently evaluated the psychometric properties of translated versions of self-administered PROMs, which are used in patients affected by RC disease and glenohumeral instability. According to the authors, when it comes to RC disorders, the only available disease-specific questionnaire for Italian patients is the translated version of the RC-QOL [41]. Unfortunately, as concluded by the authors, the poor methodological quality of the study makes the translated version unsuitable for its clinical use.

A rigorous methodological quality is surely the main strength of the present study. Although the Italian version of the DASH [32] is routinely used and many of the items are similar to the WORC items, the DASH is not specific for RC disorders. It includes items relating not only to conditions other than RC disease, but also to other joints of the upper extremity and some of the questions are subsets of other questions, such as the severity of arm/shoulder/hand pain when performing a specific activity. In this perspective, the Italian version of the WORC filled an important gap.

The present study has also some shortcomings. First, responsiveness has not been tested yet. However, considering the similar results of the psychometric properties between the Italian and the original as well as the other translated version, also a similar responsiveness could be assumed for the Italian version with caution until further clinical studies confirm the hypothesis. Moreover, the sample size in the present study was too limited to perform a factor analysis. Considering that the factor analysis of the original version [6], as well as the Canadian French version [23], did not support the five-domain organization, same results could be reasonably expected for the Italian version. Until further studies clarify the issue, only the global score should be used, as the different sections could not represent different factors.

In conclusion, the Italian version of the WORC questionnaire is a valid and reproducible measuring instrument. It has kept unchanged the concepts expressed in the original version, adapting them with Italian culture. For this reason, the WORC questionnaire can be considered a valid tool for the evaluation of the effectiveness of a treatment in terms of quality of life, in Italian patients affected by RC diseases.

### Supplementary Information

Below is the link to the electronic supplementary material.

Supplementary file1 (DOC 65 KB)
