# Supplementary material for: Cross-cultural adaptation and validation of the Italian version of the Western Ontario Rotator Cuff (WORC) index
Source: Musculoskelet Surg. 2024 Jan 29;108(2):173–81. doi: 10.1007/s12306-023-00812-y (PMC11133139; doi:10.1007/s12306-023-00812-y)
Supplement: Supplementary file 1 — Supplementary file1 (DOC 65 KB) [file 12306_2023_812_MOESM1_ESM.doc]

**WESTERN ONTARIO ROTATOR CUFF INDEX**

**(WORC)**

ISTRUZIONI PER I PAZIENTI

Nel seguente questionario ti sarà chiesto di rispondere a delle domande nel modo seguente e tu dovrai dare la tua risposta mettendo una barra “/” sulla linea orizzontale.

ESEMPIO

1. Se metti una barra “/” al limite sinistro della linea i.e.

Allora stai indicando che non hai dolore.

1. Se metti una barra “/” al limite destro della linea i.e.

Allora stai indicando che il tuo dolore è estremo.

1. Si ricorda:
2. Che più a **destra** metti la tua barra “/”, **più** provi quel sintomo.
3. Che più a **sinistra** metti la tua barra”/”, **meno** provi quel sintomo.
4. **Per favore non metter la barra al di fuori dei limiti della linea**.

In questo questionario ti è chiesto di indicare, l’entità del sintomo che hai provato nella scorsa settimana riferibile al tuo problema alla spalla. Se non sei sicuro riguardo la spalla coinvolta o hai altre domande, per favore chiedi prima di compilare il questionario.

Se per qualche ragione non capisci una domanda, per favore fai riferimento alle spiegazioni che puoi trovare alla fine del questionario. Puoi quindi mettere la tua barra “/” sulla linea orizzontale sul punto appropriato. Se una voce non ti riguarda o non hai provato quel sintomo nella scorsa settimana, per favore ipotizza la risposta che ritieni sarebbe la più accurata.

**SEZIONE A: Sintomi fisici**

**ISTRUZIONI PER I PAZIENTI**

Le seguenti domande riguardano i sintomi fisici che hai provato a causa del tuo problema alla spalla. In tutti i casi, per favore, inserisci l’importanza del sintomo che hai provato nella scorsa settimana.(per favore segna la tua risposta con una barra “/”)

1. Quanto dolore acuto provi alla tua spalla?

nessun dolore
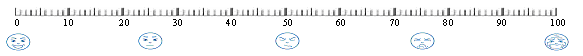
dolore estremo

1. Quanto dolore costante e fastidioso provi alla tua spalla?

nessun dolore
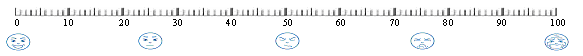
 dolore estremo

1. Quanta debolezza provi alla tua spalla?

nessuna debolezza
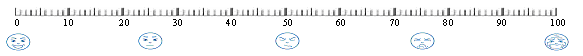
debolezza estrema

1. Quanta rigidità o perdita di range di movimento provi alla tua spalla?

nessuna rigidità
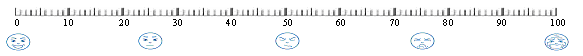
estrema rigidità

1. Quanto sei infastidito da scatti, crepitii o scricchiolii alla tua spalla?

nessun fastidio
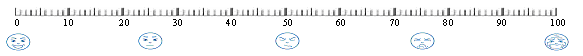
 estremo fastidio

1. Quanto disturbo provi nei muscoli del collo a causa della tua spalla?

nessun disturbo
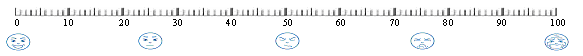
 disturbo estremo

**SEZIONE B: Sport/Attività ricreative**

**ISTRUZIONI PER I PAZIENTI**

La sezione seguente riguarda il modo in cui il tuo problema alla spalla ha influito sulle tue attività sportive e ricreative nella scorsa settimana. Per ciascuna domanda, per favore segna la tua risposta con una barra “/”.

1. Quanto la tua spalla ha influito sul tuo livello di forma fisica?

non ha influito
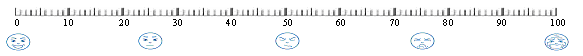
ha influito estremamente

1. Quanta difficoltà provi a causa della tua spalla nel fare flessioni o esercizi per di spalla faticosi?

nessuna difficoltà
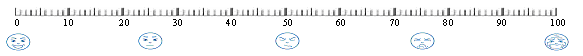
estrema difficoltà

1. Quanto la tua spalla ha influito sulla tua abilità a lanciare lontano o con forza?

non ha influito
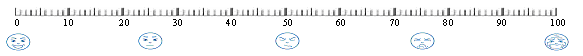
ha influito estremamente

1. Quanto sei preoccupato che qualcuno o qualcosa colpisca la tua spalla affetta?

nessuna preoccupazione
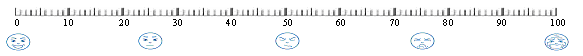
 preoccupazione estrema

**SEZIONE C: Lavoro**

**ISTRUZIONI PER I PAZIENTI**

La seguente sezione riguarda quanto il tuo problema alla spalla ha influito sul tuo lavoro in casa o fuori casa. Per favore indica la risposta più appropriata con una barra “/”, riferendoti alla scorsa settimana.

1. Quanta difficoltà provi nelle attività quotidiane in casa o in giardino?

nessuna difficoltà
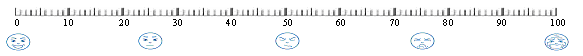
 estrema difficoltà

1. Quanta difficoltà provi lavorando al di sopra la tua spalla?

nessuna difficoltà
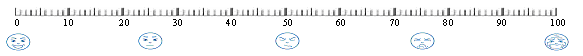
estrema difficoltà

1. Quanto utilizzi il braccio sano per compensare quello affetto?

per niente
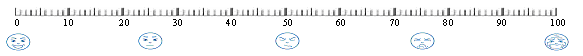
 costantemente

1. Quanta difficoltà provi ad alzare oggetti pesanti al livello o sotto il livello della spalla?

nessuna difficoltà
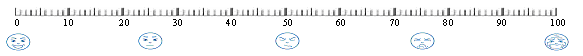
estrema difficoltà

**SEZIONE D: Stile di vita**

**ISTRUZIONI PER I PAZIENTI**

La seguente sezione riguarda quanto il tuo problema alla spalla ha influenzato o cambiato il tuo stile di vita. Per favore, indica la risposta più appropriata con una barra “/”, riferendoti alla scorsa settimana .

1. Quanta difficoltà hai a dormire a causa della tua spalla?

nessuna difficoltà
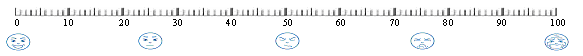
estrema difficoltà

1. Quanta difficoltà hai provato nel pettinarti i capelli a causa della tua spalla?

nessuna difficoltà
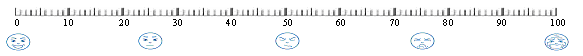
estrema difficoltà

1. Quanta difficoltà hai a scherzare e fare a botte per gioco con i tuoi familiari o amici?

nessuna difficoltà
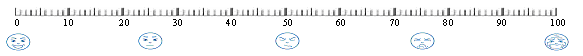
estrema difficoltà

1. Quanta difficoltà hai a vestirti o a spogliarti?

nessuna difficoltà
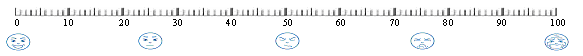
 strema difficoltà

**SEZIONE E: Emozioni**

**ISTRUZIONI PER I PAZIENTI**

Le seguenti domande riguardano come ti sei sentito durante la scorsa settimana a causa del tuo problema alla spalla. Per favore segna la risposta con una barra “/”.

1. Quanta frustrazione provi a causa della tua spalla?

nessuna frustrazione
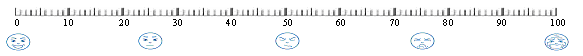
estrema frustrazione

1. Quanto ti senti “giù di morale” o depresso a causa della spalla?

per niente
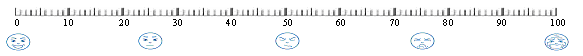
 estremamente

1. Quanto sei ansioso o preoccupato riguardo l’effetto che la tua spalla può avere nella tua attività lavorativa?

per niente
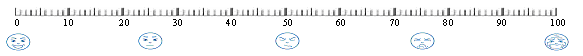
estremamente preoccupato

GRAZIE PER AVER COMPLETATO IL QUESTIONARIO

Spiegazione sul significato delle domande nel Western Ontario Rotator Cuff Index

**Sezione A : Sintomi Fisici**

Domanda 1.

Si riferisce a un dolore nella tua spalla che è rapido e improvviso o che potresti riferire come una morsa.

Domanda2.

Si riferisce ad un dolore sordo, di fondo che sembra esserci sempre, al contrario del dolore acuto di cui si parla alla domanda 1.

Domanda3.

Si riferisce alla mancanza di forza nel compiere un movimento.

Domanda4.

Si riferisce alla sensazione che l’articolazione non voglia muoversi. Questa si prova spesso al mattino quando ti alzi, dopo esercizio o dopo un periodo di inattività. Potrebbe inoltre riferirsi alla sensazione di non avere il movimento completo della tua spalla in tutte o in alcune direzioni.

Domanda5.

Si riferisce a qualunque di questi rumori o sensazioni che tu provi nella tua spalla con qualunque tipo di movimento.

Domanda6.

Si riferisce a quanta tensione, dolore o spasmo che provi nei muscoli del tuo collo che sembrano essere causati dal tuo problema alla spalla.

**Sezione B : Sport/Attività ricreative**

Domanda7.

Si riferisce al livello di forma fisica che tu mantenevi prima che la tua spalla diventasse un problema. Incluso una diminuizione del tono o della forza muscolare, dell’efficienza cardiovascolare.

Domanda8.

Si riferisce a qualunque esercizio che ti richieda di fare forza sulla tua spalla come flessioni, panca piana ecc.

Domanda 9.

Si riferisce a qualunque attività al di sopra della testa che ti richiede una certa forza nell’esecuzione. Se non lanci una palla, per favore considera qualunque altra attività come schiacciare a pallavolo, lanciare un bastoncino al tuo cane, nuotare a stile libero, servire a tennis ecc

Domanda10.

Per favore pensa a tutte le volte in cui hai avuto il sospetto o paura che qualcuno o qualcosa colpisse o entrasse in contatto con la tua spalla affetta ad esempio durante una attività sportiva, in una stanza affollata, in un ascensore o con una pacca sulla spalla durante un saluto.

**Sezione C: Lavoro**

Domanda11.

Si riferisce ad attività come rastrellare, spalare, passare l’aspirapolvere, spolverare, strappare le erbacce, zappare, lavare finestre o i pavimenti ecc.

Domanda12.

Si riferisce a qualunque attività che ti richieda di portare le braccia al di sopra del livello della spalla, come ad esempio: riporre i piatti nella credenza, cercare di prendere un oggetto, dipingere un soffitto o qualcosa al di sopra del livello della spalla, etc.

Domanda13.

Ti chiede se ora utilizzi l’altro braccio per qualunque attività o lavoro che generalmente avresti svolto con il braccio del lato affetto. Se anche l’altra spalla è sintomatica per una patologia di cuffia o qualche altra patologia, allora considera come avresti risposto se quella spalla fosse normale.

Domanda14

Questa non si riferisce al sollevamento di oggetti sopra la testa, ma al sollevamento di qualunque oggetto pesante sotto il livello della spalla come ad esempio: la borsa della spesa, una confezione di bibite, una valigia, strumenti da lavoro, libri, etc.

**Sezione D: Stile di vita**

Domanda15.

Si riferisce alla necessità di cambiare la posizione in cui dormi, svegliarsi nella notte, avere difficoltà ad addormentarsi o svegliarsi come se non avessi riposato.

Domanda16.

Si riferisce a qualsiasi cosa tu voglia fare con i tuoi capelli come pettinarli, spazzolarli o lavarli e che ti richieda di sollevare il tuo braccio affetto.

Domanda17.

Si riferisce a qualunque tipo di attività di gioco brusca o pesante che normalmente faresti con la tua famiglia o con gli amici.

Domanda18.

Si riferisce alla capacità di arrivare dietro la schiena per tirare su o giù una cerniera, abbottonare o sbottonare dei bottoni, allacciare o slacciare il reggiseno, mettere o togliere un maglione o una maglia sopra la testa, o infilarsi una camicia o una maglietta.

**Sezione E : Emozioni**

Domanda 19.

Si riferisce alla frustrazione che provi a causa della tua incapacità a fare cose che normalmente fai o che vorresti fare ma non puoi.

Domanda20.

“Essere giù di morale” o depresso non ha bisogno di altre spiegazioni.

Domanda21.

Si riferisce all’ansia che la tua spalla peggiori anzichè migliorare o che resti la stessa e alla preoccupazione circa l’effetto che questo avrà sulla tua occupazione o lavoro (considera sia il lavoro dentro che fuori casa).
